# Patients experience satisfaction and less initial waiting time after implementation of an Emergency Department with an observation unit

**DOI:** 10.1186/1757-7241-21-S2-A38

**Published:** 2013-09-09

**Authors:** Maria Søe Mattsson, Hanne B Jørsboe

**Affiliations:** 1 Emergency Department, Nykøbing F. hospital, Denmark

## Background

Patients’ experiences are an import guide to improvements in developing an Emergency Department (ED). Therefore, in 2009, when the ED at Nykoebing Hospital was established, a large group of patients were interviewed few hours after arrival. They generally expressed high degree of satisfaction with the treatment. Now, three years after, a comparable population has been interviewed to follow up after the comprehensive intervention of building up an ED. Special attention was given to the overall satisfaction with the treatment, supplemented with specific questions about initial waiting time and cooperation among staff.

## Methods

Interventional study based on structured interviews with a questionnaire containing 15 validated questions with a 5-point scale - some related to LUP, supplemented with department-specific questions. The study was initiated in 2009 and repeated again in 2012. The interventions were a package of measures. The inclusion criteria were; patient seeking ED; 18 years old or older; oriented and were able to give informed consent. Patient were triaged as orange, yellow or green and admitted to the ED for a minimum of two hours. Chi-square (chi^2^) for significance testing and a confidence interval of 95% was used.

## Results

Three years after establishment of the ED, 95% of the patients (N=579, 293 M, 286 W, mean age 63) where either satisfied or very satisfied with the treatment, which compares to the study in the very early days of the ED (N=388, 188 M, 195 W, mean age 64). Data shows a significant improvement in the patients’ experience of initial waiting time, 14% in 2012 compared to 42% in 2009 (p<0.05). 90% of the patients perceived the same cooperation among staff groups before and after the implementation.

## Conclusion

A huge reorganization of the service of acutely ill patients had no negative impact on patients overall satisfaction. Patients experienced the same cooperation among staff groups. A benefit has been active concerning waiting time, where the study shows a significant reduction in the perceived initial waiting time.

